# The Air We Breathe: Air Pollution as a Prevalent Proinflammatory Stimulus Contributing to Neurodegeneration

**DOI:** 10.3389/fncel.2021.647643

**Published:** 2021-06-24

**Authors:** Monika Jankowska-Kieltyka, Adam Roman, Irena Nalepa

**Affiliations:** Department of Brain Biochemistry, Maj Institute of Pharmacology, Polish Academy of Sciences, Krakow, Poland

**Keywords:** particulate matter, neurotoxicity, neuroinflammation, oxidative stress, neurodegeneration, autoimmunity

## Abstract

Air pollution is regarded as an important risk factor for many diseases that affect a large proportion of the human population. To date, accumulating reports have noted that particulate matter (PM) is closely associated with the course of cardiopulmonary disorders. As the incidence of Alzheimer’s disease (AD), Parkinson’s disease (PD), and autoimmune disorders have risen and as the world’s population is aging, there is an increasing interest in environmental health hazards, mainly air pollution, which has been slightly overlooked as one of many plausible detrimental stimuli contributing to neurodegenerative disease onset and progression. Epidemiological studies have indicated a noticeable association between exposure to PM and neurotoxicity, which has been gradually confirmed by *in vivo* and *in vitro* studies. After entering the body directly through the olfactory epithelium or indirectly by passing through the respiratory system into the circulatory system, air pollutants are subsequently able to reach the brain. Among the potential mechanisms underlying particle-induced detrimental effects in the periphery and the central nervous system (CNS), increased oxidative stress, inflammation, mitochondrial dysfunction, microglial activation, disturbance of protein homeostasis, and ultimately, neuronal death are often postulated and concomitantly coincide with the main pathomechanisms of neurodegenerative processes. Other complementary mechanisms by which PM could mediate neurotoxicity and contribute to neurodegeneration remain unconfirmed. Furthermore, the question of how strong and proven air pollutants are as substantial adverse factors for neurodegenerative disease etiologies remains unsolved. This review highlights research advances regarding the issue of PM with an emphasis on neurodegeneration markers, symptoms, and mechanisms by which air pollutants could mediate damage in the CNS. Poor air quality and insufficient knowledge regarding its toxicity justify conducting scientific investigations to understand the biological impact of PM in the context of various types of neurodegeneration.

## Introduction

Air pollution is regarded as an important risk factor for many diseases that affect a large proportion of the human population (Mannucci et al., 2015). The most frequent and pronounced associations are elicited by the negative consequences of polluted air within the respiratory system, causing or worsening the course of chronic obstructive pulmonary disease, asthma, or allergic reactions. Depending on the exposure conditions, source of particles, their chemical composition, and their physical properties, their health effects may differ (Wyzga and Rohr, 2015). Short-term exposure (from several hours to several days) to high concentrations of ambient particulate matter (PM) may trigger acute reactions in an organism, primarily in the most vulnerable population groups, i.e., children, pregnant women, the elderly, and people suffering from chronic disorders (Anderson et al., 2012; Mannucci et al., 2015; Kurt et al., 2016; Morakinyo et al., 2016; Sanidas et al., 2017). Long-term exposure even to relatively low concentrations of air pollutants over many years contributes to chronic diseases (Chen et al., 2008a), increasing mortality due to respiratory and cardiovascular incidents (Anderson et al., 2012; Kurt et al., 2016; Morakinyo et al., 2016; Sanidas et al., 2017), lung cancer morbidity (Li et al., 2018), reproductive function disorders (Carré et al., 2017) and postnatal development alternations (Sram et al., 2017). Air pollution is also a prevalent detrimental stimulus to the central nervous system (CNS) that has been slightly overlooked as one of many plausible causes of neuroinflammation processes. During the last few years, several research groups have considered PM exposure as an important environmental risk factor for neurotoxicity that may potentiate the risk of neurodevelopmental and neurodegenerative disorders (Block and Calderón-Garcidueñas, 2009; Ranft et al., 2009; Levesque et al., 2011; Genc et al., 2012). Nevertheless, to date, the suggested mechanism responsible for air pollution-induced pathology in the CNS or the consequent activation of multiple deleterious pathways has not been completely explored and validated.

### The Matter of Composition

Airborne particulate matter comprises a complex mixture of chemical and biological constituents. The composition of PM can substantially vary across geographical regions, sources of emissions, and even weather or seasons (Cheung et al., 2011; Chen R. et al., 2017). Particles from natural sources originate from minerals (soil and sea salt), biogenic agents (plant pollen, spores, and microorganisms), sandstorms, volcanic eruptions, earthquakes, and wildfires. However, the prevailing anthropogenic sources of PM include power station emissions, heating fumes (soot and ash), and dust from roads and construction work (Kim et al., 2015; Wang et al., 2017). The predominance of anthropogenic particles is mostly related to industry, transportation, and fuel combustion (e.g., motor vehicle emissions, traffic-related exhaust, and particles from tire and brake wearing; Karagulian et al., 2015; Wang et al., 2017). Primary particles are directly emitted from their sources into the atmosphere. Secondary particles, which stem from photochemical reactions and physical processes in the atmosphere, concomitantly increase the risk posed by air pollution (Kelly and Fussell, 2012). Several reports concerning the negative effects of PM emphasize its dependence on the chemical composition, groups of compounds, surface properties (e.g., charge and primary or secondary coatings), and specific elements after both short-term and long-term exposure (Wang et al., 2017). The carbonaceous part of air pollution is regarded as more involved in adverse health effects, and some polycyclic aromatic hydrocarbons (PAHs) are considered particularly important (Castaño-Vinyals et al., 2004; Wyzga and Rohr, 2015). However, several elements and inorganic components (Al and S) are also responsible for the detrimental effects on human health (Kim et al., 2015). The major metal constituents of PM include Cr, Co, Ni, Mn, Zn, V, Cu, and mainly Fe. The latter is present in particularly high concentrations in pollution produced by fossil fuel combustion (Park et al., 2006).

### The Matter of Size

It has been acknowledged that the size of particles is directly associated with the main causes of the negative impacts of PM (Brown et al., 2013). Ambient particles characterized by size are divided into coarse particles (PM10) with diameters of 2.5–10 μm, fine particles (PM2.5) with diameters below 2.5 μm, submicron particles (PM1) with diameters less than 1 μm, and ultrafine particles (UFPM or PM0.1) smaller than 100 nm (Block and Calderón-Garcidueñas, 2009). The minor size fraction of PM is the main contributor to the particles’ high chemical and biological activity both locally and systemically due to their large aggregated surface area (Cassee et al., 2013). Given their greater particulate mass, large particles do not remain well suspended in air aerosol. Smaller particles can be sustained in the air for a prolonged time and achieve orders of magnitude higher particulate counts and surface areas, allowing greater adsorption of other toxic air pollutants (Allen et al., 2017).

### The Matter of Adsorption

The increasing toxicity of PM depends more on the particle surface area than its composition (Sager et al., 2008). More specifically, significantly greater conveyance of adsorbed components is allowed by the larger surface of UFPM (Block and Calderón-Garcidueñas, 2009; Mazzoli-Rocha et al., 2010). The literature data reveal that ultrafine particles elicit the release of some cytokines and free radicals, which is a phenomenon not observed after exposure to corresponding fine particles. In these studies, using the same dose, ultrafine particles were 41 times more toxic than fine particles on the 1st day after exposure. However, when the dose was normalized by the surface of the administered particles, this relation decreased to two (Singh et al., 2007; Sager et al., 2008). Notably, some adsorbed compounds (e.g., transition metals and lipopolysaccharides) may become toxic stimuli independent of the particle or undergo multiple interrelated chemical reactions that may further increase toxicity (Peters et al., 2006; Block and Calderón-Garcidueñas, 2009). Thus far, researchers appear to only have a preliminary understanding of the factors in air pollution that may play a significant role in CNS pathology.

## Routes of Pm Entering and Affecting The Brain

**Figure 1:**
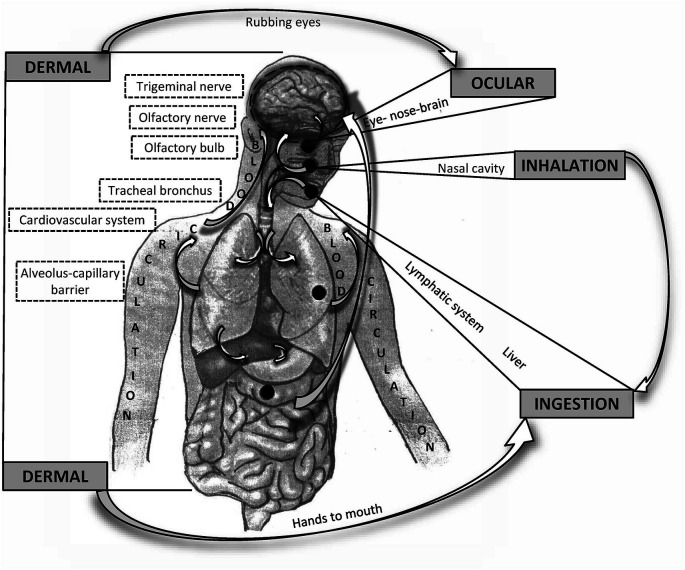
Schematic overview of possible routes of particle matter (PM) entering and affecting the brain. General routes are bolded in capital letters. Potential interactions between the different pathways are indicated by arrows.

When considering the influence of PM on the CNS, whether particles reach the brain remains unclear. The most apparent impact of air pollution occurs in the respiratory system and through the respiratory system (Anderson et al., 2012). A significant portion of pollutant masses, mainly large particles (PM10), is captured and removed in the upper respiratory tract *via* mucociliary clearance, mechanical processes (coughing and sneezing), or engulfment by macrophages. The smallest particles (PM0.1) enter the alveoli, where they can pass directly into the blood vessels. From the circulatory system, particles may cross the blood-brain barrier (BBB) to the CNS. Some of the smallest particles can penetrate directly into the brain from the nasal or oral cavity through the olfactory epithelium, olfactory nerve, trigeminal nerve, and vagal afferents (Oberdörster et al., 2004). A significant portion of particles eliminated from the upper respiratory tract is swallowed and absorbed through the gastrointestinal (GI) tract (Mutlu et al., 2018). The potential route through the eyes remains still not sufficiently clarified but it cannot be tacitly ignored (Boyes et al., 2012). All of these routes and processes also seem to be involved in the pathomechanisms of mental and neurodegenerative diseases (Figure 1).

### From the Periphery to the Brain (or Not)

It has been demonstrated that residues of airborne particles deposited throughout the alveolar region of the lung can translocate across the alveolar-capillary barrier into the systemic circulation and subsequently to extrapulmonary organs, including the brain (Oberdörster et al., 2004; Elder and Oberdörster, 2006). The CNS is an immunologically privileged area. It is isolated by a functional tight BBB that prevents the penetration of pathogens, leakage of toxins, and peripheral pathological factors, including inflammatory mediators. The BBB is built and maintained by blood vessel endothelial cells called pericytes, contiguous and interconnected with astrocytes, basal lamina, and microglia (Liebner et al., 2018). Those cell-cell adhesion complexes acting as paracellular gates and membrane fences are described as tight junctions (TJs). Under physiological conditions, the transport of essential nutrients into the brain and disposal of waste products are based on relevant specialized transporter systems. Despite the presence of this specific barrier that separates the brain from the systemic circulation, some evidence in the literature suggests that blood-derived particles may translocate through the intact BBB and increase its permeability (Sharma et al., 2009). Several nanoparticles, including manganese, iridium, silver, titanium, and cerium dioxides, have been detected in different brain areas, which is noticeably suggestive of the translocation and wide brain distribution of particles upon inhalation conditions in rodents (Elder and Oberdörster, 2006; Yokel et al., 2013; Kreyling, 2016; Patchin et al., 2016). Without an exactly recognized mechanism, it is presumed that particles are able to trigger local and systemic inflammation, leading to the release of inflammatory mediators and cytokines. TLR4/NADPH oxidase-dependent mechanisms are presumed to be involved (Kampfrath et al., 2011; Woodward et al., 2017). Activation of TLR2/NF-kB has also been demonstrated, both in vascular endothelial cells (Le et al., 2019) and in neuronal cells (Tian et al., 2021). In general, the TLR signaling activation results in the production of inflammatory cytokines and interferons (type 1) that occurs through a MyD88-dependent and interleukin-1 receptor (TRIF)-dependent pathways. Importantly, the genes encoding TLRs (TLR1–10) in humans are present in polymorphic variants that may contribute to the appearance of malfunctioning proteins and lead to increased susceptibility to autoimmune diseases (Zhang et al., 2021). Combined with excessive reactive oxygen species (ROS) generation by activated glia, further oxidative stress, alternations in the mitochondrial potential, and decreased viability of brain microvascular endothelial cells, particles could presumably induce TJ leakage and eventually interrupt the BBB even without crossing it (Choi et al., 2014; Mumaw et al., 2016).

### Nose-to-Brain Route

In inhalation studies, a direct route by which nanosized PM arrives at the nasal mucosa through the cribriform plate along the olfactory nerve axon and subsequently reaches the olfactory bulb (OB) has been recently documented (Matsui et al., 2009; Lucchini et al., 2012; Ajmani et al., 2016). Subsequently, nanoparticles target the olfactory cortex by afferent olfactory neurons (Wang et al., 2017). Similarly, after nasal instillation of ultrafine particles, translocation to the OB and induction of tumor necrosis factor alpha (TNFα) and macrophage inflammatory protein (MIP)-1α were noted (Win-Shwe et al., 2015b). It has been proven that inhaled UFPM rapidly causes oxidative stress in the olfactory epithelium. Subsequent inflammatory and neurodegenerative responses in OB include increased apoptosis in olfactory neurons. Delayed responses in the cerebral cortex and cerebellum involve the increased expression of TNFα (Cheng et al., 2016). This particular route is of special interest in terms of neurodegenerative aspects since OB neurons are among the first to display characteristic markers of Parkinson’s disease (PD).

### Eye-to-Brain Way

Besides, a fraction of airborne PM could be inhaled through the nasal mucosa, it could also enter the eye socket directly through the air in dust-polluted areas or be transferred from the hands through rubbing of the eyes (Wang et al., 2017). Direct contact of nanoparticles with the eye might lead to absorption and penetration into the ocular tissues, however, a tear film with aqueous and lipid layers and the corneal epithelium with tight junctions may limit this penetration. Although the retina has a blood-retinal barrier that is similar to the BBB, particles small enough to cross the BBB might be expected to easily enter the retina (Seyfoddin et al., 2010). Thus, there is some unclear probability that particles can drain into the nasal cavity through the nasolacrimal duct and subsequently enter the CNS (Boyes et al., 2012). According to advanced pharmaceutical research conducted with nanomaterials to explore enhanced penetration following eye drop application (Nagpal et al., 2010; Seyfoddin et al., 2010), particles with selective solubility and nanosized parameters can penetrate the eye after contact exposure.

### The Microbiome-Gut-Brain Axis

The previously described routes of PM entry into the brain demonstrate that some of the smallest particles can be translocated through sensory nerve endings (Jayaraj et al., 2017). A significant fraction of particles eliminated from the upper respiratory tract via mucociliary clearance is swallowed with saliva and mucus and may be absorbed in the GI tract (Möller et al., 2004; Beamish et al., 2011). In the guts, air pollution components have a negative effect on intestinal flora by altering its natural composition and inducing a chronic pro-inflammatory tendency in the body *via* ROS generation and nuclear factor NF-kB activation (Salim et al., 2014; Mutlu et al., 2018). Several reports indicate that in addition to chronic inflammation, PM exposure causes an increase in gut permeability associated with a disruption of tight junction proteins in the colonic epithelium (Mutlu et al., 2011). GI epithelial cells act as the primary physical barrier of the gut, and this barrier function might be affected by the intestinal epithelial injury induced by PM (Beamish et al., 2011; Salim et al., 2014). Although it is not possible to quantify how much PM exactly reaches the GI tract, recent emerging research suggests that the microbiota–gut–brain axis is also involved in neurodegenerative diseases, such as Alzheimer’s disease (AD) and PD, and psychiatric conditions, such as addiction (Sherwin et al., 2018; Stefano et al., 2018). Accumulating evidence supports the view that gut microbes influence central neurochemistry and behavior and are capable of producing most neurotransmitters found in the human brain (Dinan and Cryan, 2017). Gut bacteria are able to contact the brain through the vagus nerve and immune system and through the production of microbial metabolites (e.g., cresol) and modulation of circulating tryptophan (O’Mahony et al., 2015; Foster et al., 2016; Sarkar et al., 2016). In reference to another neurodegenerative disorder with an autoimmune background, it has been recently demonstrated that altered gut microbiota can inhibit the expression of myelin genes and differentiation of oligodendrocytes, certainly providing a new perspective regarding the pathogenesis of multiple sclerosis (MS; Hoban et al., 2016).

## Potential Mechanism Underlying The Particle-Induced Detrimental Effects on The Cns

Despite the large diversity in the chemical composition and physical properties of air pollutants, the most frequently described toxicity mechanisms are the induction of oxidative stress and inflammatory processes in the periphery and CNS (Wang et al., 2017). Both mechanisms are tightly linked, interdependent, and regarded as key players in neurodegeneration pathogenesis (Jellinger, 2010; Fagundes et al., 2015). Among the potential mechanisms underlying the particle-induced detrimental effects on the CNS and the etiology of neurodegenerative disease, increased oxidative stress, neuroinflammation, endoplasmic reticulum (ER) stress, mitochondrial dysfunction, and disturbance of protein homeostasis are often postulated (Wang et al., 2017).

### Oxidative Stress and Neuroinflammation

Highly reactive free radicals, particularly ROS in the form of superoxide anion (${\text{O}}_{2}^{-}$), are produced in mitochondria as a result of respiratory chain activity (Pavlin et al., 2016; Angelova and Abramov, 2018). At low concentrations, free radicals in some immune cells (monocytes, macrophages, and granulocytes) regulate functioning and participate in intracellular signaling and the elimination of microorganisms absorbed by phagocytosis in a mechanism of cytotoxicity (Sarniak et al., 2016; Tan et al., 2016). There are numerous enzymatic and nonenzymatic antioxidative processes in cells that maintain an oxidoreductive balance. Both organic and inorganic particles can generate ROS directly on their surface as a response to a cell or a whole organism. Free radicals produced as a result of particle-cell interactions implicate lipid peroxidation, nucleic acid damage, and structural disruption of proteins. Transition metals present in PM act as catalysts in Fenton’s reactions and serve as a source of ROS, whereas the organic fraction induces the expression of CYP450 cytochrome (CYP1A1) and enhances ROS production with the involvement of xenobiotic biotransformation enzymes (Pavlin et al., 2016; Wang et al., 2017). The oxidative changes induced by free radicals in biologically important molecules, including proteins, lipids, and nucleic acids, may lead to a loss of their functionality (Pavlin et al., 2016; Angelova and Abramov, 2018). Even small disturbances in the functioning of the respiratory chain or antioxidant mechanisms may initiate oxidative stress, contributing to the further escalation of the process via mitochondrial damage and impairment of their function. Mitochondria are, in fact, simultaneously the main source of free radicals and the first target of their negative action. These organelles contain lipid membranes, enzymatic proteins, and mitochondrial DNA, being extremely important for life processes and are susceptible to oxidative damage by free radicals formed in their immediate vicinity (Angelova and Abramov, 2018; Nissanka and Moraes, 2018).

Within the overall response of the body to air pollution, oxidative stress and inflammatory processes are closely related. In the lungs, particles are absorbed (phagocytized) by pulmonary macrophages and other phagocytes. As a result of phagocytosis, cell activation and the activation of intracellular mechanisms of ROS synthesis used for intracellular killing of pathogens occur (Sarniak et al., 2016). Intensive ROS generation causes a change in the oxidoreductive status of macrophages and their proinflammatory activation (Tan et al., 2016). Nevertheless, it is necessary to consider the fact that immune processes play undeniably vital roles in maintaining neuroprotection and regeneration (Hammond et al., 2019). However, this evoked pathway involves the release of numerous inflammatory mediators that activate the immune system (proinflammatory cytokines) and cell migration factors (chemokines) that increase the expression of adhesion molecules in various cell types, including immune cells and endothelial blood vessels. As a result, this pathway may lead to unsealing the blood-brain barrier and the inflow of peripheral cells of the immune system to the brain, which is among the pathomechanisms of the inflammatory process in the CNS (Chen et al., 2016). Thus, the peripheral inflammatory processes induced by air pollution may be transferred to the CNS and participate in neurodegeneration. Similar phenomena occur in the CNS under the influence of particles penetrating along nerves. In the CNS, brain macrophages, i.e., microglia cells, are activated analogously. This activation occurs in response to the presence of particles in the brain or as a reaction to incoming inflammatory signals from the periphery (Chen et al., 2016). Differing from other cells, neurons are excitable cells that require additional energy to maintain proper membrane potential and generate and conduct action potentials and the intracellular transport of neurotransmitters (Pavlin et al., 2016). Intensive ROS synthesis in the immediate vicinity of neurons leads to a vicious cycle of oxidative stress, cellular energy metabolism modulation, impairment of their function, and, in extreme situations, death (Peixoto et al., 2017). A high content of nucleic acids, proteins, and unsaturated fatty acids and a low concentration of antioxidants render the brain more vulnerable to redox imbalance. It is well known that oxidative stress and inflammatory processes in the CNS are the basis of the typical phenomena observed in neurodegeneration. Therefore, interference with neurodegenerative processes by the effects of air pollution is likely as indicated in clinical and experimental studies (Angelova and Abramov, 2018; Nissanka and Moraes, 2018).

### Glial Activation and BBB Damage

Microglia constitute a special population of tissue macrophages in the CNS that represent up to 16% of cells (depending on the structure of the brain) and perform various functions as follows: participate in creating and removing synapses, detecting and fighting infections, eliminating damaged cells and tissue deposits, repairing nervous tissue and restoring normal function (Roman et al., 2013). Microglial cells undergo proinflammatory activation by endogenous cell-damaging factors or peripheral proinflammatory signals. Thus, the peripheral activation of the immune system and inflammatory processes can induce analogous processes in the CNS, especially in cases of impaired BBB permeability (Liebner et al., 2018). The proinflammatory activation of microglial cells is associated with a change in their morphology, increases in the expression of many surface markers, and release of migration mediators (chemokines), facilitating an influx of peripheral immune cells to the CNS, the release of free radicals (oxygen and nitrogen species) and synthesis of cytotoxic cytokines, such as TNF-α, interleukin-1 beta (IL-1β) and interferons (Roman et al., 2013). These processes tend to remove pathogens or damaged cells, further extinguish inflammation, rebuild tissue, and restore its normal function. Under pathological conditions, uncontrolled proinflammatory activation of microglia can lead to secondary damage to cerebral vascular cells and the escalation of this phenomenon. Astroglia is another cell type in the brain that plays crucial roles in BBB integrity, providing contact with neurons, maintaining homeostasis of ions, moderating excess neurotransmitters, and secreting neurotrophic factors (Seifert et al., 2006). Almost all types of injuries within the CNS result in an astroglial activation response (Chen et al., 2016). In humans chronically exposed to high levels of air pollution, astroglia is reportedly activated as confirmed by the enhanced expression of glial fibrillary acidic protein (GFAP), which is a marker of astrocytic activation, in the amygdala and frontal cortex (Calderón-Garcidueñas et al., 2004, 2008b; Allen et al., 2014b). Whether astroglia responds to air pollution components, inflammation, oxidative stress processes, or cellular damage remains questionable. PM has also been suggested to have the ability to both interact with the cells that form the BBB and penetrate the BBB through insufficiently identified mechanisms. Presumably, nanosized particles have the capacity to injure endothelial cells and damage the BBB by decreasing brain microvascular endothelial cell viability, disturbing the mitochondrial potential, sustaining oxidative stress, and decreasing the expression of TJ proteins (Chen L. et al., 2008). The response of the cerebral vasculature to air pollution exposure may manifest as an increase in the cell adhesion molecules (intracellular, ICAM; vascular, VCAM), the production of cytokines or ROS and the upregulation of efflux transporters (P-glycoprotein and multidrug resistance-associated protein-2), having significant implications for drug availability in the brain parenchyma for individuals living in heavily polluted cities and contributing to CNS pathology. Inflammatory processes play an important role in the pathomechanisms of neurodegeneration, including both those generated in the periphery and those occurring in the CNS. MS is an example of such an occurrence; in MS, the immune response to the myelin sheath peptides of nerve fibers is induced in the periphery, and this process is transferred to the CNS, eventually causing neurodegeneration (Kawachi and Lassmann, 2017). Direct proinflammatory activation of microglia could also be a result of neuronal damage caused by oxidative stress and cellular energy deregulation and the presence of deposits of abnormal proteins or infectious agents and toxins (von Bernhardi et al., 2015; Chen et al., 2016). Oxidative stress also activates microglial cells because the oxidoreductive state of the microenvironment and the presence of ROS affect the functional status of macrophages (Tan et al., 2016). Microglial activation and inflammatory processes are commonly observed in the CNS in the course of many neurodegenerative diseases and the natural aging process and are considered among the most important or earliest pathomechanism components (von Bernhardi et al., 2015; Chen et al., 2016; Kawachi and Lassmann, 2017).

### Alternations in Protein Homeostasis

Many late-onset neurodegenerative diseases are characterized by the formation of intra- or extracellular protein aggregates. The extracellular plaques in AD mainly consist of amyloid β (Aβ), and the neurofibrillary tangles are formed by hyperphosphorylated tau proteins. In PD, the intracellular inclusions (Lewy bodies) are composed of alpha-synuclein (Bellucci et al., 2012; Breydo et al., 2012). Currently, the abnormal aggregation of misfolded proteins in neurodegeneration is thought to originate from genetic mutations, posttranslational modification, malfunctions of mitochondria or the ER, calcium imbalance, or excitotoxic glutamatergic overstimulation (Agorogiannis et al., 2004; Goodwin et al., 2013). Accumulated damaged or misfolded proteins under physiological conditions are successfully removed by cell clearance based on autophagy. In the case of the failure of this mechanism, inflammatory reactions and oxidative damage may be triggered. The direct or indirect effect of inhalable particles on protein homeostasis is still under consideration. Although, the association among PM composed of metals, oxidative stress, inflammation processes mediated by damage to mitochondria or proteins, and the intensification of protein aggregation in PD and AD provides a potential, interesting link between air pollution and neurodegeneration. Combining protein aggregation in neurodegeneration processes with environmental factors deserves more attention, and in this context, it is worth mentioning that, the formation of aggregates in AD and PD is not limited to the brain (Calingasan et al., 2005). During the early stages of these diseases, protein aggregates are found in the spinal cord and peripheral nervous system, which notably broadens views regarding the pathological basis of neurodegeneration (Braak et al., 2003; Clairembault et al., 2015).

### Importance of Glymphatic System

The glymphatic system includes the subarachnoid space and the system of perivascular spaces penetrating deep into the brain along with blood vessels, filled with cerebrospinal fluid (CSF). The movement of the fluid forces the constant production of CSF in the choroid plexus as well as the heart rate, vascular pulsation, and, to a lesser extent, respiratory movements. This system plays a very important role in the removal of harmful metabolites, including Aβ, tau protein and α-synuclein, tissue fluid replacement, and nutrient delivery (Jessen et al., 2015). Studies conducted in recent years have shown disturbances in the flow of CSF in the glymphatic system in patients with AD (Tuovinen et al., 2020). These alterations were mainly caused by the weakening of intracranial cardiovascular pulsation and correlated with the severity of cognitive dysfunction. Hypertension causes a significant reduction in the flow of the CSF in the glymphatic system, which reduces the leaching of harmful metabolic products, including Aβ (Mestre et al., 2018). As exposure to PM has the greatest negative impact on the respiratory and cardiovascular systems (Kim et al., 2017; Ain and Qamar, 2021), also indirectly influences the glymphatic system, contributing to the accumulation of incorrectly folded proteins in the brain, which may intensify neurodegenerative changes. This indirect effect of PM on the glymphatic system and neurodegenerative processes is still poorly understood and is the subject of further research, especially since the non-invasive assessment of the glymphatic system function using the fMRI method may allow for a relatively early diagnosis of neurodegenerative disorders, including AD (Tuovinen et al., 2020).

### Disturbances in the Dopamine and Glutamate Systems

According to the available data, PM exposure appears to alter both neurotransmitters within dopamine and glutamate systems. Some reports address the issue of glutamatergic disturbances, although notably, the pattern of changes is not consistent. A significant body of research provides evidence of elevated Glu levels in the hypothalamus after prenatal 4-week inhalational exposure of adolescent male mice to PM or an increase in the mRNA expression of the NMDA receptor subunits NR1, NR2A, and NR2B in the hippocampus (Win-Shwe et al., 2008, 2015a). However, a 3-month exposure of female adolescent mice did not result in any changes in NMDA receptor subunits and was merely accompanied by decreased levels of the glutamate transporter EAAT4 in the mentioned brain area (Win-Shwe et al., 2012a). Another study in female mice with highly comparable exposure and the same structure uncovered an increase in the levels of NR2A mRNA expression at higher doses (Win-Shwe et al., 2012b). Elsewhere, as a result of 10-week inhalation exposure of mice to nanosized PM, the neuronal glutamate receptor subunit (GluA1) was decreased in the hippocampus. In turn, in hippocampal slices after 2 h exposure, those researchers observed increased GluA1, GluN2A, and GluN2B, but not GluA2, GluN1, or mGlur5 post-synaptic proteins in cornu ammonis area-1 (CA1) neurons. These findings further document the impact of PM on glutamatergic functions (Morgan et al., 2011; Davis et al., 2013).

A similar situation applies to dopamine. Some researchers describe a decrease in the level of the dopamine metabolite homovanillic acid (HVA) and dopamine turnover in the striatum, nucleus accumbens, or brainstem following prenatal exposure to air pollution (Yokota et al., 2009). However, scientists have also reported reductions in the level of dopamine and its metabolites in the prefrontal cortex and amygdala, which were evident at 3 weeks but not 6 weeks later (Yokota et al., 2013). Furthermore, in the subsequent work conducted by this group, prenatal inhalation exposure to diesel exhaust increased dopamine and its metabolites in the prefrontal cortex and nucleus accumbens but not in the amygdala and ventral tegmental area (Yokota et al., 2016). In another study, alterations in dopamine turnover were observed in the striatum (Suzuki et al., 2010).

Based on previously available research and collected data, it is difficult to draw a coherent conclusion regarding this potential mechanism of the negative impact of air pollution. It is even more difficult to connect the observed changes under the premises of PM influence on neurodegenerative processes in this regard. The differences in the obtained results may be due to the heterogeneity of the PM materials used in the exposure treatment, differences in the experimental design and life span of animals, the exposure method, the same-sex approach to research, or additional factors and stimuli, such as behavioral tests with varying degrees of invasiveness to animals.

### Genetics and Epigenetics

The relationship between genetic mutations and neurodegenerative diseases is repeatedly emphasized. Interestingly, regarding neurodegeneration, only a few genes have been considered causative agents, but most genes are described as susceptibility-increasing genes (Burbulla and Krüger, 2011; Rohn, 2013; Lee and Cannon, 2015). An example of the latter type is the apolipoprotein E gene (*ApoE*), whose variants are basically linked to cardiovascular disorders and further AD (Giau et al., 2015). The link between genes and the environment has been investigated in terms of some ApoE carriers, who appear to be more affected by air pollution than individuals without a mutation (Calderón-Garcidueñas et al., 2015; Schikowski et al., 2015). Similarly, in familial PD, the gene locus of leucine-rich repeat kinase 2 (*LRRK2*) mutations is considered to be involved in sensitivity to environmental insults instead of being causative for PD (Lee and Cannon, 2015). Susceptibility to environmental factors is specified as a feature related to epigenetic changes, especially in neurodevelopmental abnormalities. The utmost issue encountered in this matter is the difficulty in establishing such interactions because exposure to an environmental insult early in life may only result in subtle epigenetic changes, which, in turn, could have a relevant effect in the form of increased susceptibility to age-related neurodegenerative disorders subsequently in life (Modgil et al., 2014). Some reports demonstrated that exposure to lead (Pb) during the early life period causes a temporary upregulation of *APP* (b-amyloid precursor gene in AD) in neonates, but the levels are normalized in adulthood. It was observed that subsequently in aged animals developmentally exposed to Pb, *APP* was increased again. Most interestingly, exposure of animals only in their late age did not result in similar changes in *APP* gene expression (Basha et al., 2005; Zawia et al., 2009). This type of research strengthens the phenomenon of ’genetic imprinting’, which is construed as a trace from environmental insult in childhood that subsequently increases vulnerability to age-related disorders. Although it seems very likely that PM exposure should be considered as such a negative environmental impact, more research is required to draw conclusions regarding this issue.

## Air Pollution, Neurodegenerative Processes, and Cns Diseases

Neurodegeneration is a pathological condition characterized by the loss of functions, structure, or both within the nervous system or neurons (Fakhoury, 2016). Neurodegeneration leads to neuronal death and is referred to as a predominantly irreversible process. The most common neurodegenerative diseases are Alzheimer’s disease and PD. Neurodegeneration also accompanies many other diseases of the nervous system, such as multiple sclerosis, depression (Brown et al., 2018), stress-related disorders (Miller and Sadeh, 2014), anxiety disorders (Perna et al., 2016), and autism (Kern et al., 2013). Neurodegenerative changes are also associated with the natural aging process (Wyss-Coray, 2016). Generally, these processes tend to appear later in life and are characterized by the progressive dysfunction or loss of specific neuron populations, which determines the clinical picture of the disease. Behaviorally, neurodegeneration may manifest as cognitive impairment along with motor and somatic disabilities. At the anatomical level, various structural changes are found in specific areas of the brain accompanied by functional and biochemical alterations. The extracellular or intracellular accumulation of abnormal proteins is reportedly a characteristic histological hallmark. Despite differences in the clinical symptoms of many diseases, dysfunction and death of neurons are closely related to the disease course and are based on common neurodegeneration mechanisms (Jellinger, 2010). These mechanisms include abnormal protein synthesis and degradation, oxidative stress, impairment of mitochondrial functioning and cellular energy metabolism, induction of inflammatory processes, and microglial activation. The formation of intracellular protein deposits hinders the essential process of intracellular transport in neurons, resulting in abnormal neurotransmission. Protein aggregates also contribute to mitochondrial dysfunction and excessive ROS synthesis, resulting in impaired neuronal functions and death [36]. Mechanisms enabling the removal of abnormally shaped or structured proteins supposedly maintain homeostasis, but their malfunctioning leads to cell death and represents the main pathomechanism of various neurodegenerative diseases, including AD and PD (Jeong, 2017; Zeng et al., 2018).

**Figure 2:**
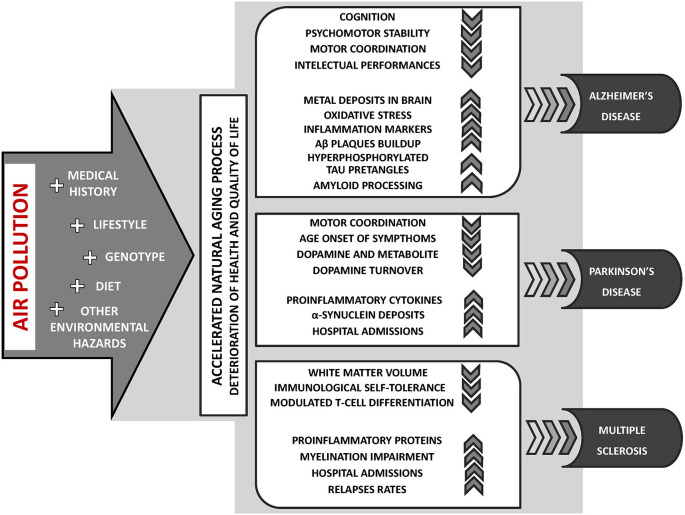
The key aspects of air pollution exposure contribution to the incidence or course exacerbation of neurodegenerative diseases: Alzheimer’s disease (AD), Parkinson’s disease (PD), and multiple sclerosis (MS).

Currently, an escalating body of research provides evidence suggesting that exposure to air pollution may contribute to the incidence or course exacerbation of neurodegenerative diseases, including AD (Babadjouni et al., 2017; Kilian and Kitazawa, 2018), PD (Ritz et al., 2016; Lee et al., 2017) and MS (Roux et al., 2017; Ashtari et al., 2018; Figure 2). Furthermore, long-term exposure to air pollution is significantly related to the earlier occurrence of aging symptoms, mild cognitive impairment (MCI), and dementia (Clifford et al., 2016; Allen et al., 2017; Babadjouni et al., 2017; Sram et al., 2017).

### Air Pollution as a Risk Factor for AD

Alzheimer’s disease is the most common form of dementia, affecting over 50 million patients worldwide (International Alzheimer’s Disease, 2019). AD and other dementias are currently considered the sixth of the top 10 causes of death globally (The Top 10 Causes of Death, 2020). The estimates of the world’s largest health organizations suggest that these numbers may double or triple in the next few decades. Alzheimer’s disease is clinically characterized by the loss of memory, inability to learn and calculate, loss of language function, a deranged perception of space, indifference, depression, delusions, and other manifestations. AD is progressive and fatal within approximately 4–8 years from diagnosis depending on other factors (Stages of Alzheimer’s. n.d. Alzheimer’s Disease and Dementia, 2020). This neurodegenerative disorder is driven by the following two processes: extracellular deposition of insoluble beta amyloid—Aβ (a component of senile plaques) and intracellular accumulation of insoluble hyperphosphorylated tau protein (the component of neurofibrillary tangles). Common pathogenic theories of AD and aging emphasize the concept that lesions and protein deposits are the initiators of the disease due to their toxicity. Aβ deposition is specific to AD and is thought to be primary. Another early pathological hallmark of AD that is strongly correlated with cognitive impairment is synapse loss in the neocortex and hippocampus. This synapse loss is most likely due to Aβ oligomers acting on multiple synaptic NMDA-type glutamate receptors and α7-nicotinic acetylcholine receptors (Jeong, 2017). The role of neuroinflammation, free radicals, diet, genetics, excitotoxicity, nonenzymatic glycation of proteins, and other factors contributing to the loss of neurons and synapses in the etiology of AD is highly important and actively explored (Atri, 2019). AD pathology is indicative of an active host response, adaptation, or a response to environmental factors that may contribute to disease onset and progression (Castellani and Perry, 2014). The most frequently mentioned and proven risk factors for AD include aging and genetic predispositions, particularly the presence of the *ApoE ɛ4* allele (Raber et al., 2004). In a study involving children living in the highly polluted Mexico City, females, but not males, with one copy of the *ApoE ɛ4* allele obtained worse cognitive scores than those with homozygous *ApoE ɛ3* alleles (Calderón-Garcidueñas et al., 2016). This finding could indicate that if the genotype and environment specifically interact, certain subpopulations could be more vulnerable to the effects of air pollution on cognition. Additionally, lifestyle, medical history, dietary habits, educational background, and exposure to environmental hazards are presumed to play a key role in AD etiology (Campdelacreu, 2014; Yegambaram et al., 2015). A significant body of epidemiological research investigates how urban and rural air and distance to roadways affect brain function and cognition. Overall, the summary of these studies seems to be alarming as the one consistent message is that exposure to PM at any stage of life negatively impacts cognition (Clifford et al., 2016; Oudin et al., 2016; Xu et al., 2016).

Regarding the early-life period, living in heavily polluted areas during childhood may result in an increased risk of poor psychomotor stability and impaired motor coordination and response time (Wang et al., 2009). Compared with children living in rural areas of this country, children from the polluted Mexico City demonstrated poorer outcomes on multiple intelligence subscales, including full-scale IQ and vocabulary (Calderón-Garcidueñas et al., 2008a, 2011). By investigating the impact of individual fractions of air pollutants, it has been found that the levels of black carbons in air PM are inversely associated with some intellectual performances, including vocabulary and visual skills of learning and memory in children (Suglia et al., 2008; Chiu et al., 2013). Additionally, in postmortem tests, the metal content (manganese, nickel, and chromium) was found to be higher in the brain tissue, and the number of deposits of abnormal proteins characteristic of AD and PD, even in children and young people, was reported to be intensified (Calderón-Garcidueñas et al., 2013, 2018). Furthermore, a few studies have shown that air pollution increases the expression of oxidative stress markers and proinflammatory markers, including cyclooxygenase-2 (COX-2), IL-1β, interleukin-12 (IL-12), NF-kB, CD14, and TNF-α, and decreases the prion-related protein PrPC in multiple brain tissues in children and adults in Mexico (Calderón-Garcidueñas et al., 2004, 2008b, 2012a,b). The same group of researchers conducted extensive and pioneering research in the field of AD-specific pathology, such as the abnormal buildup of Aβ plaques, which has also been reported by others (Bhatt et al., 2015; Cacciottolo et al., 2017). Their observations indicate that children and young adults living in highly polluted areas have greater amounts of Aβ42 immunoreactivity, Aβ diffuse plaques, and hyperphosphorylated tau pre-tangles in the OB, hippocampus, and cortical neurons than control subjects (Calderón-Garcidueñas et al., 2004, 2008b, 2012b). In addition, subjects homozygous for *ApoE ɛ4* showed even more pronounced changes mentioned above than those with the *ApoE ɛ3* allele (Calderón-Garcidueñas et al., 2008b, 2012b). Undeniably, air pollution can affect neuroinflammation and amyloid processing in the human brain, which are two causes of neuronal dysfunction that precede the appearance of neuritic plaques and neurofibrillary tangles, i.e., the hallmarks of AD. However, notably, most research concerning this issue was carried out by one research group in the same region and population. This limitation does not undermine the veracity of the presented results, but additional studies with an expanded scope are needed in the future.

Most studies concerning air pollution effects on child cognition primarily pay attention to precarious surroundings and measurements of air pollution exposure, such as rural vs. city residence or distance to a roadway, rather than attributing the particular effect to an individual constituent of air pollution. Unfortunately, environmental factors other than PM may be importantly interfering considerations in determining the effects of air pollution using these types of metrics (van Kempen et al., 2012). For instance, it has been shown that road and airway noise levels negatively affect attention and cognition test scores. Considering the extended adverse effects of early-life PM exposure in the context of triggering neurodegenerative diseases subsequently in life, it could be notably challenging and desirable, if feasible, to conduct long-term studies from childhood to senescence while also considering many other factors.

*In vivo* studies examining the effects of PM exposure in an AD mouse model used female 5xFAD mice with either the *ɛ3* or *ɛ4* allele of *ApoE*. The results showed an increase in Aβ oligomers in protein samples, but only in the 5xFAD *ApoE ɛ4* mice, suggesting that PM exposure effects on AD pathogenesis can be increased in susceptible genotypes. However, reduced CA1 neuronal density in the hippocampus was observed in both the wild-type control mice and 5xFAD *ApoE ɛ3* mice with and without PM exposure (Cacciottolo et al., 2017). Another group conducting research with this mouse model observed significantly poorer outcomes in task assessing grip strength and motor coordination (but not the spatial working memory), achieved after 13 weeks of diesel engine exhaust (DEE) exposure in 5xFAD mice compared to the 5xFAD clean air exposed controls. Noteworthily, in the same task, the wild-type mice after DEE exposure also performed less well than their clean air littermates, however without reaching statistical significance. Although after 3 weeks of exposure of 5xFAD mice to DEE, none of the results in the behavioral test were significantly affected but those mice showed significantly higher cortical Aβ plaque load and higher Aβ42 protein levels than the clean air controls. Despite the sub-chronic (13 weeks) exposure to DEE, observed effects in 5xFAD transgenic mice were not mediated by systemic inflammatory responses, in analyses performed 11 days after the last exposure. Still, the possible transient inflammation during the DEE inhalation cannot be excluded, as another successful research group confirmed the differential time course of oxidative stress and inflammatory responses to nanosized PM between various parts of the brain (Cheng et al., 2016; Hullmann et al., 2017). Several *in vitro* studies have also shown a buildup of Aβ plaques as an effect of increased neuronal and glial beta-secretase (BACE) expression involved in promoting the amyloidogenic pathway of APP processing, which was correlated with a concomitant increase in the COX-1 and COX-2 protein levels and a modest alteration in the cytokine profile (Bhatt et al., 2015). In addition to neuroinflammatory states, microglia and astrocytes, which are brain resident immune cells, are involved in the metabolism and clearance of Aβ (Griffin et al., 1998; Nagele et al., 2004; Heppner et al., 2015). in vitro and *in vivo* research carried out thus far indicates that PM exposure is involved in inflammatory processes (Campbell et al., 2005), oxidative stress (Calderón-Garcidueñas et al., 2004; Zanchi et al., 2010), amyloidogenesis (Calderón-Garcidueñas et al., 2004, 2008b, 2010, 2012b; Levesque et al., 2011; Cacciottolo et al., 2017), and the negative impacts on behavior in animals (Allen et al., 2014b), but much more work involving AD animal models is needed to determine if and how PM exposure could alter AD and other dementias.

### Air Pollution as a Risk Factor for PD

PD is another progressive neurodegenerative disease with a complex etiology based on an intricate combination of environmental and genetic factors. In its course, the dominant symptoms are tremor, rigidity, and bradykinesia, and postural instability appears at a subsequent stage. PD is pathologically characterized by the loss of nigrostriatal dopaminergic innervation, although neurodegeneration is not limited to nigral dopaminergic neurons. A decisive diagnosis requires histopathological assessment with the identification of Lewy bodies containing α-synuclein proteins or dystrophic Lewy neurites. α-Synuclein aggregation is central to the development of the disease. Several other processes are thought to be involved in PD pathogenesis, and several studies suggest that abnormal protein clearance, mitochondrial dysfunction, and neuroinflammation play a crucial role in its onset and progression. The most frequently mentioned risk factors include age, genetics, and ongoing exposure to environmental toxins (Kouli et al., 2018). Exposure to air pollution could be linked to PD risk by observations of its deleterious effects on human health, most of which are related to brain inflammation and oxidative stress (Segalowitz, 2008; Block et al., 2012), which are markers thought to be relevant to the development or progression of PD (Chen et al., 2008b; Hirsch and Hunot, 2009; Ton et al., 2012). Although epidemiologic data related to this issue have been limited, some research suggests that an association exists between the risk of PD and air pollution from traffic. In a postmortem study of stray animals, children, and young adults, neuropathological lesions in feral dogs and pathologic changes similar to those in PD in olfactory neurons were found in residents living in highly polluted cities compared with individuals from rural areas. A re-examination of prior investigations in the rodent brain also indicated higher nigral levels of proinflammatory cytokines and α-synuclein deposits (Calderón-Garcidueñas et al., 2008a,b, 2010). This finding is of special importance because aggregates of the α-synuclein protein are major components of Lewy bodies, which are a PD hallmark feature. Misfolded α-synuclein proteins were recently found to be transmissible within the brain and able to spread from affected to unaffected neurons (Desplats et al., 2009; Luk et al., 2012). An early preclinical feature of PD is the loss of the sense of smell (Doty, 2012), and OB neurons are among the first to display characteristics of PD Lewy bodies according to Braak’s staging theory (Braak et al., 2004). Perhaps it is not without significance that airborne ultrafine particles have been shown to translocate through the BBB (Oberdörster et al., 2004) by first targeting afferent olfactory neurons in the olfactory cortex (Wang et al., 2017). While a few groups have conducted research investigating the increased risk of PD development after short-term and long-term exposure to air pollution, other studies have not found such associations (Lee et al., 2016; Liu et al., 2016; Ritz et al., 2016; Chen C.-Y. et al., 2017; Palacios et al., 2017). Epidemiologic research linking air pollution and dopaminergic neurodegeneration is still scarce. Although exposure to traffic-derived air pollution did not increase the PD risk (Palacios et al., 2017), there was a small growth in the PD prevalence and a lower age at onset among the participants with higher manganese (Mn) and copper (Cu) exposure (Finkelstein and Jerrett, 2007; Willis et al., 2010). The risk of PD exacerbation, which is defined as emergency hospital admission for primarily diagnosed PD, was significantly increased following short-term exposure to higher concentrations of air pollutants, including PM2.5, NO_2_, SO_2_, and CO (Lee et al., 2017). Additionally, the PD prevalence has been associated with annual increases in airborne metal concentrations (Palacios et al., 2014), PM10 and PM2.5 (Liu et al., 2016) and long-term exposure over 20 years to NO_2_ (Ritz et al., 2016) among female never smokers. The results of other studies reporting the PD risk reveal statistically significant associations with NOx, NO_2_, CO, and O_3_, and the most significant association was between the PD risk and CO. However, exposures to PM2.5, PM2.5–10, PM10, and SO_2_ were not found to be significantly related (Hu et al., 2019). Nevertheless, the absence of an association does not necessarily imply the absence of a causal relationship. Differences in population demographics, study design, exposure method or duration, and variation in the accuracy of regional air pollution monitoring may be responsible for these inconsistent results, indicating the need for conducting more comprehensive studies.

### Air Pollution as a Risk Factor for Multiple Sclerosis

Multiple sclerosis is a generalized degenerative disease of the brain and spinal cord in which neuronal loss and atrophy of the brain tissue occur as a result of the following two basic pathological processes: inflammation and neurodegeneration. Inflammation is represented by myelin-specific T lymphocytes directed against the myelin protein of myelin sheaths surrounding axons. Autoreactive T lymphocytes sensitized to myelin components may lead to the increased expression of chemotactic cytokines and adhesion molecules and the migration of T lymphocytes or macrophages to the CNS white matter. The activation of the cascade of immune system protein reactions leads to the development of inflammation and numerous areas of demyelination. The formation of such areas can also be initiated by the death of oligodendrocytes, followed by secondary axon damage, gradually contributing to neurodegeneration. Most likely, both processes occur in a manner dependent on each other, but which process initiates the disease remains unknown. Depending on the location of the damaged area in the CNS, symptoms, such as visual disturbances, paresis, balance problems, speech and muscle tension disturbances, dizziness, fatigue, thermal hypersensitivity, and perception disorders, may occur but usually not simultaneously. Although most patients exhibit remissions between relapses, MS is described as a heterogeneous disease without a typical clinical course pattern (Hauser and Oksenberg, 2006).

While the mechanism of disease progression is increasingly familiar, the cause of the disease remains unclear. An association between genetic and environmental factors and autoimmunity in MS has been found. Even though the exact cause triggering relapses in MS is not well known, a strong linkage between elevated concentrations of pollutants in the air (PM10, SO_2_, NO_2_, and NO_x_) and increased susceptibility to infections has been observed, and some pollutants are positively correlated with relapses in MS patients (Oikonen et al., 2003; Kaźmierski et al., 2004; Ascherio and Munger, 2007). Similarly, studies performed in Georgia, US suggest a potential role of PM10 in the etiology of MS, especially in females. However, notably, this large study was based on self-declared diagnosis of MS (without confirmation by a neurologist) and unclear measures of air pollution while supposing that its levels were equivalent over several years. A group of Iranian researchers also found that long-term exposure to high levels of the mentioned air pollutants caused higher relapse rates and differentiated MS prevalence. Furthermore, the results of a French study showed a significant association between the risk of relapse and PM10 levels within 3 days prior to relapse. Another group of French researchers cautiously concluded that PM10 exposure appears to be a trigger for MS relapse. These authors rightly note a certain dependence of seasonal relapse variation and season-dependent factors, such as meteorological parameters and air pollution. Finally, researchers in Italy supported the hypothesis that high levels of PM10 may play a role in determining MS occurrence, relapses, and hospital admissions (Gregory et al., 2008; Heydarpour et al., 2014; Leray et al., 2015; Vojinović et al., 2015; Angelici et al., 2016; Roux et al., 2017). Additionally, a few imaging studies revealed a reduction in the white matter volume and myelination impairment as a consequence of exposure to PM and linked constituents of air pollution (Prado et al., 2018). Biochemical tests have shown the presence of antibodies against some neuronal proteins and myelin in the blood (Babadjouni et al., 2017).

Overall, thus far, only a few studies have attempted to examine the impact of air pollution on MS relapses, and all studies have a few methodological weaknesses questioning the results. Nevertheless, the mechanism by which this particular factor may affect the disease has not been clarified. However, MS relapses seem to be initiated by inflammation, which raises its importance as a neurological model disease to study the influence of air pollution. One hypothesis assumes that exposure to PM causes the secretion of proinflammatory proteins and oxidative factors unsealing the BBB, leading to immune attack mediated by activated microglial cells and resulting in neuroinflammation. Another hypothesis suggests that air pollution could decrease immunological self-tolerance by producing autoantibodies or modifying the gut microbiota, limiting the delivery of UVB and causing vitamin D deficiency (Esmaeil Mousavi et al., 2017). Despite the lack of conclusive significant evidence and numerous extensive studies in this field, it is worth considering air pollutants as among the noteworthy reasons for MS relapses and exacerbation of symptoms.

*In vivo* studies examining the effects of PM exposure in an MS mouse model used experimental autoimmune encephalomyelitis (EAE) mice with comparable lesions as in the case of MS patients induced by autoimmunization with brain antigens. This model is the model most commonly used to investigate this disease in terms of its pathogenesis and novel therapeutic approaches (Lassmann, 2007). Very limited research has been performed in this area in reference to the influence of PM exposure; nevertheless, some results indicate modulated T-cell differentiation and effector functions, especially in the case of Th17 and Treg cells, and identify their crucial role in the mechanisms of autoimmunity. The same diesel exhaust particles worsened the severity of EAE symptoms, but other fractions delayed the onset of EAE and reduced the peak clinical scores. Despite the small amount of and large discrepancy in the data obtained, it is postulated that exposure to PM could disorganize the balance between effector and regulatory immune cells and is responsible for Th1-mediated immunosuppression after exposure to yet unidentified components of atmospheric pollution (O’Driscoll et al., 2018, 2019).

### Air Pollution and Natural Aging

The concept of natural aging assumes a multifactorial process of molecular and cellular decline, including the loss of protein homeostasis, DNA damage, lysosomal dysfunction, epigenetic modifications, and immune deregulation, that slowly affects tissue function over time (Wyss-Coray, 2016). These complex changes lead to frailty and susceptibility to disease and death. The idea of successful aging is often understood as maintaining high cognitive and physical functions, the lack of chronic disease, and longevity (Cosco et al., 2014). Based on epidemiological research, each of these aspects may be inversely affected by chronic exposure to air pollution (Chen et al., 2008a; Baccarelli et al., 2016). Elderly individuals are more vulnerable to air pollution due to the progressive deterioration of functional properties at the molecular, cellular, tissue, and organ levels and higher prevalence of preexisting cardiovascular and respiratory diseases, resulting in higher mortality (Sacks et al., 2011; Fougère et al., 2015; Wong et al., 2015). Oxidative stress and inflammation induced by exposure to air pollution are suspected to accelerate the natural erosion of telomeres due to aging. A Belgian study confirmed that higher annual PM2.5 concentrations were linked to a decreased telomere length and reduced content of mitochondrial DNA, which are considered aging markers (Pieters et al., 2016). Both mentioned mechanisms, i.e., inflammation and oxidative damage, merge following exposure to air pollution, resulting in an increased risk of cognitive decline and dementia (Clifford et al., 2016; Seaton et al., 2020). Residents aged over 65 years who live in areas with high air pollution in China, Mexico, the USA and Germany obtained significantly lower scores on certain common cognitive tests used to assess dementia than those living further from roadways (Sánchez-Rodríguez et al., 2006; Ranft et al., 2009; Wellenius et al., 2012). The main culprit of the reduction in verbal learning and increased rate of errors in tests of working memory and orientation is considered black carbon (Power et al., 2011) and PM2.5 (Gatto et al., 2014; Ailshire and Clarke, 2015). The elevation in the overall mild cognitive impairment (MCI) incidence at a 5-year German follow-up exam in a cohort aged 47–75 years was significantly related to the latter (Tzivian et al., 2016). Every 10 μg/m^3^ increase in these two components is estimated to be equal to an additional two years of cognitive decline by aging (Power et al., 2011). Notably, no similar relationship was found between PM exposure and cognitive decline in a younger adult population (mean age of approximately 37 years; Ranft et al., 2009). *In vitro* and *in vivo* studies are indispensable to decipher the link between exposure to PM and the development of dementia. A few reports have demonstrated that short-term exposure (2–6 weeks) to high levels of ultrafine PM in mice can elicit significant increases in proinflammatory cytokines, i.e., IL-1α and TNF-α, glial responses, and the activation of NF-kB and AP-1 transcription factors in brain tissue (Campbell et al., 2005; Kleinman et al., 2008). Similarly, 6–10 weeks of exposure to PM ranging from nanoscale to PM2, 5 in C57BL/6 mice and Wistar rats resulted in increased IL-1α, IL-1β, TNFα, heme oxygenase-1 (HO-1), GFAP, CD14, and CD68 mRNA, which together confirm increased brain inflammation (Guerra et al., 2013; Cheng et al., 2016). Unsurprisingly, after long-term exposure to PM2.5 (30–39 weeks) in mice, the dominant effect was also the inflammatory response in the brain with an additional significant loss of dendritic spine density and dendrite length in the CA1 region of the hippocampus, which is related to impaired cognitive outcomes (Bhatt et al., 2015). Recent *in vitro* studies revealed that high levels of PM applied to murine microglial BV2 cells or rat-derived mixed astrocyte/microglia culture resulted in cytotoxicity and increased the secretion of proinflammatory cytokines (TNF-α, IL-6, and iNOS mRNA; Morgan et al., 2011; Cheng et al., 2016). Concomitant with increased inflammation in hippocampal slice culture, PM exposure evoked concentration-dependent NMDA receptor-mediated neurotoxicity (Morgan et al., 2011). Although the levels of multiple neurotransmitters and cytokines are often reported to be altered following exposure to PM, sometimes there are significant changes and large variances between groups and sexes (Allen et al., 2014a, b).

Although neurodegenerative disorders affect several proportions of the global population, aging affects everyone; it is tempting to classify neurodegenerative diseases as manifestations of accelerated pathological aging. However, this oversimplification does not accurately reveal the underlying mechanisms that tie these two issues. Instead, to enhance our understanding of how aging contributes to disease, it is worth considering how environmental factors and genes interact in particular diseases with distinct hallmarks of aging and identifying the importance of these processes in neurodegenerative processes (Wyss-Coray, 2016).

## Concluding Remarks

**Figure 3:**
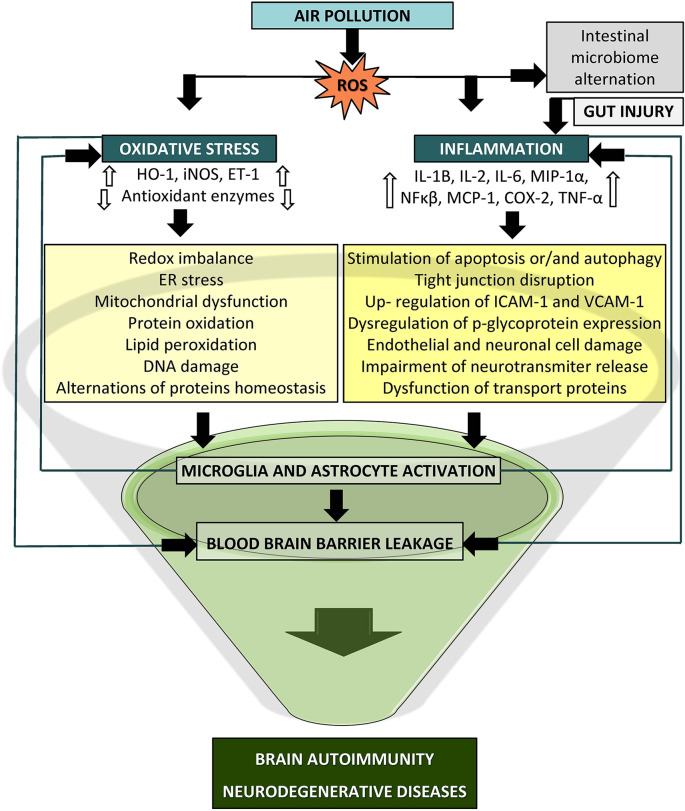
Potential mechanism of particle-induced detrimental effects on the central nervous system (CNS). Oxidative stress and inflammation may lead to blood brain barrier (BBB) breakdown via activated immune cascades, redox imbalance, mitochondrial dysfunction, and microglia and astrocyte activation which could culminate in brain autoimmunity and neurodegeneration (COX-2, cyclooxygenase2; ET-1, endothelin1; HO-1, heme oxygenase1; IL, interleukin; iNOS, inducible nitric oxide synthase; MCP-1, monocyte chemoattractant protein1; MIP1-a, macrophage inflammatory protein-1α, NF-κB, nuclear factor kappa B; TNF-α, tumor necrosis factor alpha).

This article provides an overview of the current views and knowledge regarding the impact of air pollution on the cellular processes responsible for the emergence of oxidative stress and inflammation that may eventually lead to autoimmune responses and neurodegenerative diseases (Figure 3). Previous research provides a strong foundation for the case of air pollutants as adverse factors for neurodegenerative disease etiologies. The strongest premises concern the role of inflammation and oxidative processes as a common ground shared by air pollutants and neurodegeneration. Nevertheless, there is much inconsistency in the effects reported in the presented studies, which is most likely a consequence of various sources and concentrations of pollutants, different times of exposure, testing paradigms, and endpoints, which altogether may elicit diversified and novel toxicity affecting the brain. The understanding of the neurotoxic effects of air pollution and its constituents is still limited, and previous investigations have several discrepancies; hence, further studies are needed to comprehend the cellular and molecular basis underlying the relationship between air pollution and neurodegeneration processes. Further research should determine the relevance of the applied measurement methodology, exposure conditions and extended duration, additional environmental and anthropogenic factors, and animal models for humans in relation to relevant exposure situations. There should be still an emphasis on the identity of the specific neurotoxic mechanisms. A more comprehensive approach with higher adequacy to humans supporting the translation of the findings into public health regulations seems to be an important issue to advance this field of research in the future. Certainly, we must be circumspect in our conclusions. PM exposure may not be responsible for damage to a specific brain region or a given disease but rather could induce a challenging state in the brain that then manifests according to an individual’s condition.

## Author Contributions

MJ-K wrote the manuscript. AR and IN reviewed and edited the manuscript. All authors contributed to the article and approved the submitted version.

## Conflict of Interest

The authors declare that the research was conducted in the absence of any commercial or financial relationships that could be construed as a potential conflict of interest.
